# Inhibition of Fatty Acid Synthase Upregulates Expression of CD36 to Sustain Proliferation of Colorectal Cancer Cells

**DOI:** 10.3389/fonc.2020.01185

**Published:** 2020-07-31

**Authors:** James Drury, Piotr G. Rychahou, Daheng He, Naser Jafari, Chi Wang, Eun Y. Lee, Heidi L. Weiss, Bernard Mark Evers, Yekaterina Y. Zaytseva

**Affiliations:** ^1^Department of Toxicology and Cancer Biology, University of Kentucky, Lexington, KY, United States; ^2^Markey Cancer Center, University of Kentucky, Lexington, KY, United States; ^3^Department of Surgery, University of Kentucky, Lexington, KY, United States; ^4^Department of Pathology and Laboratory Medicine, University of Kentucky, Lexington, KY, United States

**Keywords:** lipogenesis, anticancer activity, FASN-targeted therapy, fatty acid metabolism, TVB inhibitors

## Abstract

Fatty acid synthase, a key enzyme of *de novo* lipogenesis, is an attractive therapeutic target in cancer. The novel fatty acid synthase inhibitor, TVB-3664, shows anti-cancer activity in multiple cancers including colorectal cancer; however, it is unclear whether uptake of exogeneous fatty acids can compensate for the effect of fatty acid synthase inhibition. This study demonstrates that inhibition of fatty acid synthase selectively upregulates fatty acid translocase (CD36), a fatty acid transporter, in multiple colorectal cancer models including colorectal cancer cells with shRNA mediated knockdown of fatty acid synthase and genetically modified mouse tissues with heterozygous and homozygous deletion of fatty acid synthase. Furthermore, human colorectal cancer tissues treated with TVB-3664 show a significant and selective upregulation of CD36 mRNA. shRNA-mediated knockdown of CD36 and inhibition of CD36 via sulfosuccinimidyl oleate, a chemical inhibitor of CD36, decreased cell proliferation *in vitro* and reduced tumor growth in subcutaneous xenograft models. Isogenic cell populations established from patient derived xenografts and expressing high levels of CD36 show a significantly increased ability to grow tumors *in vivo*. The tumor-promoting effect of CD36 is associated with an increase in the levels of pAkt and survivin. Importantly, combinatorial treatment of primary and established colorectal cancer cells with TVB-3664 and sulfosuccinimidyl oleate shows a synergistic effect on cell proliferation. In summary, our study demonstrates that upregulation of CD36 expression is a potential compensatory mechanism for fatty acid synthase inhibition and that inhibition of CD36 can improve the efficacy of fatty acid synthase-targeted therapy.

## Introduction

Colorectal cancer (CRC) is the leading cause of non-smoking related cancer deaths in the world (1). Altered fatty acid metabolism is a hallmark of cancer and a potential target for therapeutic intervention (2–4).

Fatty Acid Synthase (FASN), a key enzyme of *de novo* lipogenesis, is significantly upregulated in CRC and promotes tumor growth and metastasis (5–7). Novel FASN inhibitors developed by Sagimet Biosciences show anti-cancer activity in lung, prostate, ovarian, and colon cancer models *in vitro* and *in vivo* (8–10), and are currently being tested in phase I/II clinical trials (11–13). Our studies show anti-tumor activity of TVB inhibitors in primary CRC cells and CRC patient-derived xenograft (PDX) models (10, 14).

While most tumors exhibit a shift toward FA synthesis, they can also scavenge lipids from their environment (4). Fatty Acid Translocase (CD36), a multifunctional glycoprotein, has an important role in fatty acid metabolism as a fatty acid receptor and transporter (15, 16). CD36 translocates to the plasma membrane, where an extracellular domain of the protein binds low density lipoproteins and transports them across the plasma membrane into the cytosol, thus playing a critical role in metabolism of extracellular fatty acids (15–17). CD36 is subject to various types of post-translational modifications. Glycosylation, ubiquitination, and palmitoylation are involved in regulating CD36 stability and the rate of fatty acid uptake (18). Recent studies have shown that CD36 is highly expressed and enhances the progression of solid malignancies such as breast, ovarian, gastric, and glioblastoma cancers (19–22). Silencing CD36 in human prostate cancer cells reduces fatty acid uptake and cellular proliferation (23). Furthermore, the presence of CD36 positive metastasis initiating cells correlates with a poorer prognosis in glioblastoma and oral carcinoma (21, 24). The contribution of CD36 to CRC progression has not yet been investigated.

Since cancer cells utilize both endogenously-synthesized lipids and exogeneous fatty acids (25), and our published data indicate that an enhanced uptake of dietary fatty acids may be a potential mechanism of resistance to FASN inhibitors (10), the goal of this study was to evaluate the interconnection between these two pathways.

We found that CD36 is significantly overexpressed in CRC and that there is a correlation between expression of FASN and CD36 in primary human CRC specimens. We demonstrate that a decrease in FASN expression is associated with selective induction of CD36 and that this phenomenon is consistent among multiple cancer models. Pharmacological and shRNA-mediated inhibition of CD36 decreases proliferation of primary CRC cells *in vitro* and inhibits tumor growth *in vivo*. We also show that CD36 overexpression is associated with upregulation of survivin, a protein linked to apoptosis resistance, metastasis, bypass of cell cycle checkpoints, and resistance to therapy (26, 27). Consistent with our *in vitro* data, we show that CD36^high^-expressing cells, isolated from CRC PDXs, have a significantly higher level of survivin as compared to CD36^low^-expressing cells from the same tumor. Our results also demonstrate that combined inhibition of FASN and CD36 has a synergetic effect on inhibition of cellular proliferation suggesting that combination treatment may be a potential therapeutic strategy for CRC.

Together, our findings demonstrate the tightly regulated interconnection between *de novo* lipid synthesis and CD36-mediated lipid uptake in CRC progression during targeted inhibition of FASN, suggesting that inhibition of CD36 may be necessary to improve the efficacy of FASN-targeted therapy.

## Materials and Methods

### CRC Cell Lines

Established cell lines HCT116, HT29, and HT29LuM3 were maintained in McCoy's 5A medium supplemented with 10% FBS (Sigma-Aldrich, St. Louis, MO) and 1% penicillin–streptomycin. Primary colon cancer patient Pt 93 and Pt 130 cultures were isolated and established from PDX tumors as previously described (1). Cells were maintained as monolayer culture in DMEM supplemented with 10% FBS (Sigma-Aldrich, St. Louis, MO) and 1% penicillin–streptomycin. Primary Pt 93 and Pt 130 colon cancer cells were authenticated as unique human cell lines (Genetica). Established CRC cell lines were authenticated using STR DNA profiling (Genetica, Cincinnati, OH). Stable CD36 knockdown HCT116, HT29, and HT29LuM3 cell lines were established using CD36 shRNAs from Sigma-Aldrich (TRCN000005699, TRCN0000057000, and TRCN0000057001). Cells were selected with 10 mg/mL puromycin. Knockdown was confirmed via quantitative real-time PCR (qRT-PCR) after cell selection and prior performing animal experiments. Overexpression cell lines were established by transfecting HCT116 cells with either pCMV-Spark-CD36 (Sino Biological Inc., NM 001001547.2), td-Tomato-CD36 (Addgene, Plasmid #58077).

### Tissue Microarray Analysis—Immunohistochemistry

Immunoreactivity scores of CD36 (antibody sc-7309, Santa Cruz Biotechnology) and FASN (antibody #3180, Cell signaling) expression were analyzed in matched normal colon mucosa and tumor tissues from patients diagnosed with Stage I–IV CRC who had surgery at UK Chandler Medical Center (TMA ID BH15991A, *n* = 56) by a GI pathologist (EYL) blinded as to tumor stage. The final immunoreactivity score was determined by multiplication of the values for staining intensity (0, no staining; 1, weak; 2, moderate; 3, strong staining) and the values for percentage of positive tumor cells (0, no positive cells; 1, 0–10%; 2, 11–50%; 3, 51–100% positive).

### Tissue Collection

Tissues were obtained from consented patients with Stage II–IV CRC who had undergone surgery at UK Medical Center (IRB #16-0439-P2H). 6–8-week-old NSG mice (NOD.Cg-Prkdc Il2rg /SzJ) from The Jackson Laboratory (Bar Harbor, ME) were used for PDX models. All procedures were performed using protocols approved by the UK Animal Care and Use Committee. Briefly, CRC tissues (2–5 mm) obtained from CRC patients of both sexes were implanted subcutaneously into their flanks in a small pocket surgically created under the skin. Established tumors were designated as generation 0 (G0). Tumor tissues from G0 were minced and mixed with Matrigel to ensure homogeneous distribution of tissues among mice and allow implantation of an equal volume of tumor tissues into the flank. Tumor tissues were resected when they reached an appropriate size and digested as previously described (1). For evaluation of CD36 expression in PDX models, we utilized tissue samples from Pt 2402 PDX model established from a patient diagnosed with metastatic adenocarcinoma (lung) consistent with colon primary tumor (1). Pt 2402 PDX tumors were grown to ~200 mm^3^ then tissues were collected and lysed for analysis via western blot.

### Flow Cytometry

Individual cells from PDX model Pt 2402 were stained for CD36 with fluorescent antibody (Abcam ab23680). Stained cells were sorted via flow cytometry and the top 10% of GFP positive CD36 expressing cells (CD36^high^) and the bottom 10% of GFP negative cells (CD36^low^) were sorted separately from the rest of the tumor cell population. CD36^high^ and CD36^low^ were mixed with 100 μL of 30% Matrigel and subcutaneously injected into NSG mice. Tumor growth was monitored for three months. Samples were taken from subsequent tumors for western blot analysis and immunohistochemistry and the remaining tumor tissue was re-sorted for CD36.

### Fatty Acid Uptake

HCT116, NTC, and FASN shRNA cells were plated at 10,000 cells/well on an 8-well coverslip u-slide (Ibidi #80826) and treated with CD36 neutralizing antibody (Cayman Chemical #1009893) for 24 h. After incubation with neutralizing antibody, cells were then treated with fluorescent FA analog BODIPY FL (Thermo Fisher #D3822) for 10 min in serum free McCoy's 5A medium supplemented with 10% fatty acid free BSA. Cells were washed twice with PBS and fixed with PBS containing 5% formalin for 20 min at 37°C. Cell were then imaged via confocal microscopy using a Nikon A1 Confocal Microscope.

### Cell Proliferation Assay

CRC cell lines were plated onto 24 well-plates at a concentration of 30,000 cells per well. Cells were given DMEM medium for Pt 93 and Pt 130 and McCoy's 5A medium for HCT116 with and without FBS to simulate starvation conditions. Cells were also treated with or without 100 μM SSO (Cayman Chemical) or 0.2 μM TVB-3664 or both. TVB-3664 was provided by Sagimet Biosciences (Menlo Park, CA). SSO was purchased from Cayman Chemical (Ann Arbor, MI). Cells were incubated at 37°C for 6 days. After the incubation period, cells were trypsinized, and collected individually based on well and condition of treatment. Cells were counted using a Vi-Cell XR Cell Viability Analyzer (Beckman Coulter). HCT116, NTC, and CD36 shRNA(#2 and #4), cells were plated onto 24 well-plates at a concentration of 30,000 cells per well. Cells were cultured in McCoy's 5A medium with and without fetal bovine serum for 72 h and counted as described above.

### Quantitative Real-Time PCR

Total RNA was isolated using a RNeasy mini kit (QIAGEN). cDNA was synthesized using a high capacity cDNA reverse transcription kit (Applied Biosystems). QRT-PCR was carried out using a TaqMan Gene Expression Master Mix (#4369016) according to manufacture protocol and TaqMan probes for human CD36 (ID Hs00354519 m1), human FASN (ID Hs01005622 m1), human FATP3 (ID Hs00354519 m1), human FATP4 (Hs00192700 m1), and human GAPDH (#4333764F; Applied Biosystems).

### Subcutaneous Xenografts

NU/NU mice were injected subcutaneously with 1.0 × 10^6^ cells of HCT116 NTC (non-targeted control, *n* = 7), shCD36 #2 (*n* = 6), or shCD36 #4 (*n* = 7) in 100 μL PBS and tumor growth was monitored. Tumor size was measured via calipers every 3 days and tumor volume was calculated using the formula: TV = width^2^ × length/0.52. When NTC tumor growth reached ~200 mm^3^, all mice were sacrificed, and tumor weight was taken via digital scale. NU/NU mice were injected subcutaneously with 2.0 × 10^6^ cells of NTC (*n* = 5) and shCD36 #4 (*n* = 5) in 100 μL PBS for HT29 and HT29 LuM3 xenografts experiments.

### Genetically Modified Mice

C57BL/6J mice with LoxP-flanked FASN alleles were obtained from Clay Semenkovich, MD at Washington University, and FASN/VillinCre and FASN/Apc/VillinCre mouse colonies were established by mating these mice with C57BL/6J Villin/Cre and C57BL/6J Apc/Cre mice in Dr. Zaytseva's laboratory.

## Results

### CD36 Protein Is Overexpressed in CRC

**Figure 1:**
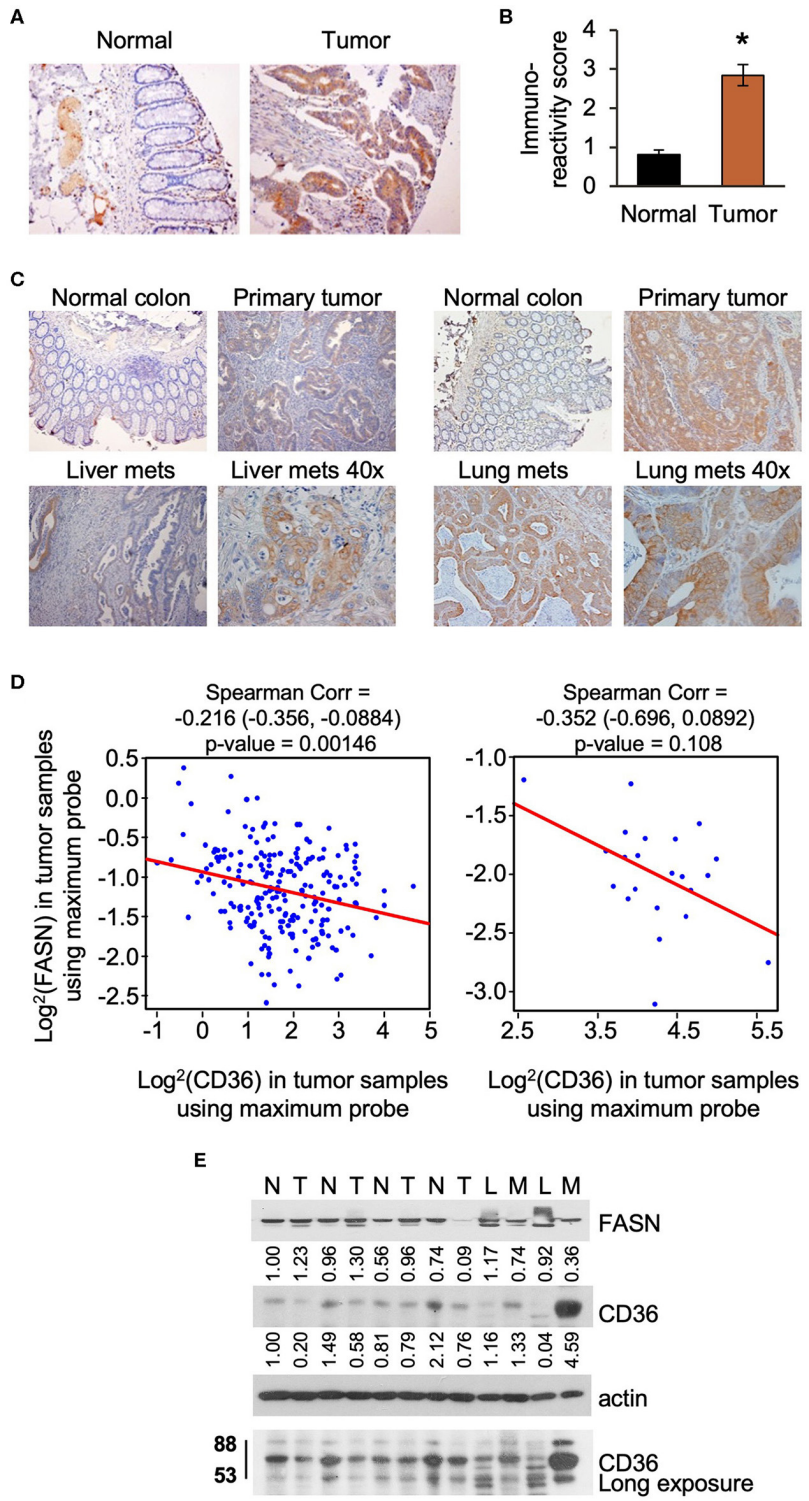
CD36 is overexpressed in human CRC. **(A,B)** Immunoreactivity score of CD36 expression was analyzed in matched normal colon mucosa and tumor tissues from patients diagnosed with Stage I–IV CRC (TMA: *n* = 56, *p < 0.001 vs. normal tissue). **(C)** CD36 staining in matched normal colon mucosa, primary CRC, and CRC metastasis to liver and lung [representative images are shown; liver (*n* = 12) and lung metastasis (*n* = 5)]. **(D)** Correlations between FASN and CD36 was determined based on RNASeq data of CRC patient samples (*n* = 22 of normal tissues and *n* = 215 of tumors) from The Cancer Genome Atlas. **(E)** Expression of FASN and CD36 in human normal colon mucosa and tumor tissues. N, normal mucosa; T, primary tumor; L, normal liver tissue; M, liver metastasis.

Upregulation of lipid metabolism is a common characteristic of many solid malignancies, and frequently, enhanced *de novo* lipogenesis occurs concomitantly with enhanced import of lipids from the extracellular space (3, 28). In our previously published study we showed that FASN is significantly overexpressed in primary tumor tissues as compared to matched normal colon mucosa using tissue microarray analysis (TMA) (29). Using the same TMA, we assessed the expression of CD36 levels in tumor tissues and found that expression was significantly higher as compared to normal colon mucosa as determined by statistical evaluation of immunoreactivity scores. We noted that the expression of CD36 is predominantly cytosolic in primary CRC tumors (Figures 1A,B). Interestingly, statistical analysis via Spearman Correlation showed a positive correlation between expression of CD36 and FASN in primary CRC tumor tissues, but it was not statistically significant (Spearman *r* = 0.21743, *n* = 56). We have also detected an increase in expression of CD36 in CRC metastasis to liver and lung (Figure 1C).

Using The Cancer Genome Atlas (TCGA) data, we also analyze FASN and CD36 mRNA expression. Consistent with protein data, the level of FASN mRNA is significantly higher in tumor tissues as compared to normal mucosa (Supplementary Figure 1A). In contrast, we found that the level of CD36 mRNA is significantly lower in cancer tissues as compared to normal tissues (Supplementary Figure 1B). Interestingly, according to data analysis from The Human Protein Atlas, the high mRNA expression of CD36 (*n* = 131) is associated with poor prognosis in CRC with 5-year survival of 53% of patients as compared to 5-year survival of 64% of patients with low CD36 mRNA expression (*n* = 466) (30). Statistical analysis of correlation between FASN and CD36 revealed a significant negative correlation between FASN and CD36 mRNA levels in tumor tissues, but not in normal tissues (Figure 1D).

To further delineate the association between expression of FASN and CD36, we analyzed the expression of these proteins in fresh human normal colon mucosa, primary CRC tissues, and metastasis (Figure 1E). The predicted molecular mass of CD36 protein is 53kD. However, due to the post-transcriptional modifications including extensive protein glycosylation, it is widely reported as ~80–88 kD protein (16, 18, 31). This size will be shown for all *in vitro* and *in vivo* data in this manuscript. In the analyzed tissues sample set, the expression of FASN is higher in primary tumors as compared to normal mucosa in most cases. Due to FASN being expressed in healthy liver tissue, it is not suprizing to see that its expression is higher in the normal liver as compared to liver metastasis. CD36 expression seems to be higher or the same in primary tumors as compared to normal colon mucosa. However, expression of CD36, particularly in its glycosylated form, is much higher in liver metastasis as compared to normal liver or normal colon mucosa (Figure 1E).

To further analyze CD36 in CRC we analyzed tissues from PDX models, which retain the intratumorally clonal heterogeneity and tumor microenvironment of the parent tumor through passages in mice (10, 32). We analyzed the expression of CD36 in nine PDXs established from primary tumors and CRC metastasis (10), and found that CD36 (88kD) is mostly associated with PDX established from metastatic tumors with the exception of Pt 2568, which was established from primary CRC tumor (10) (Supplementary Figure 1C).

Together, these data demonstrate that CD36 is upregulated and exhibits multiple post-translational modificatios in CRC and that a significant inverse correlation exists between mRNA expression of FASN and CD36 in primary human CRC.

### FASN Selectively Regulates Expression of CD36

**Figure 2:**
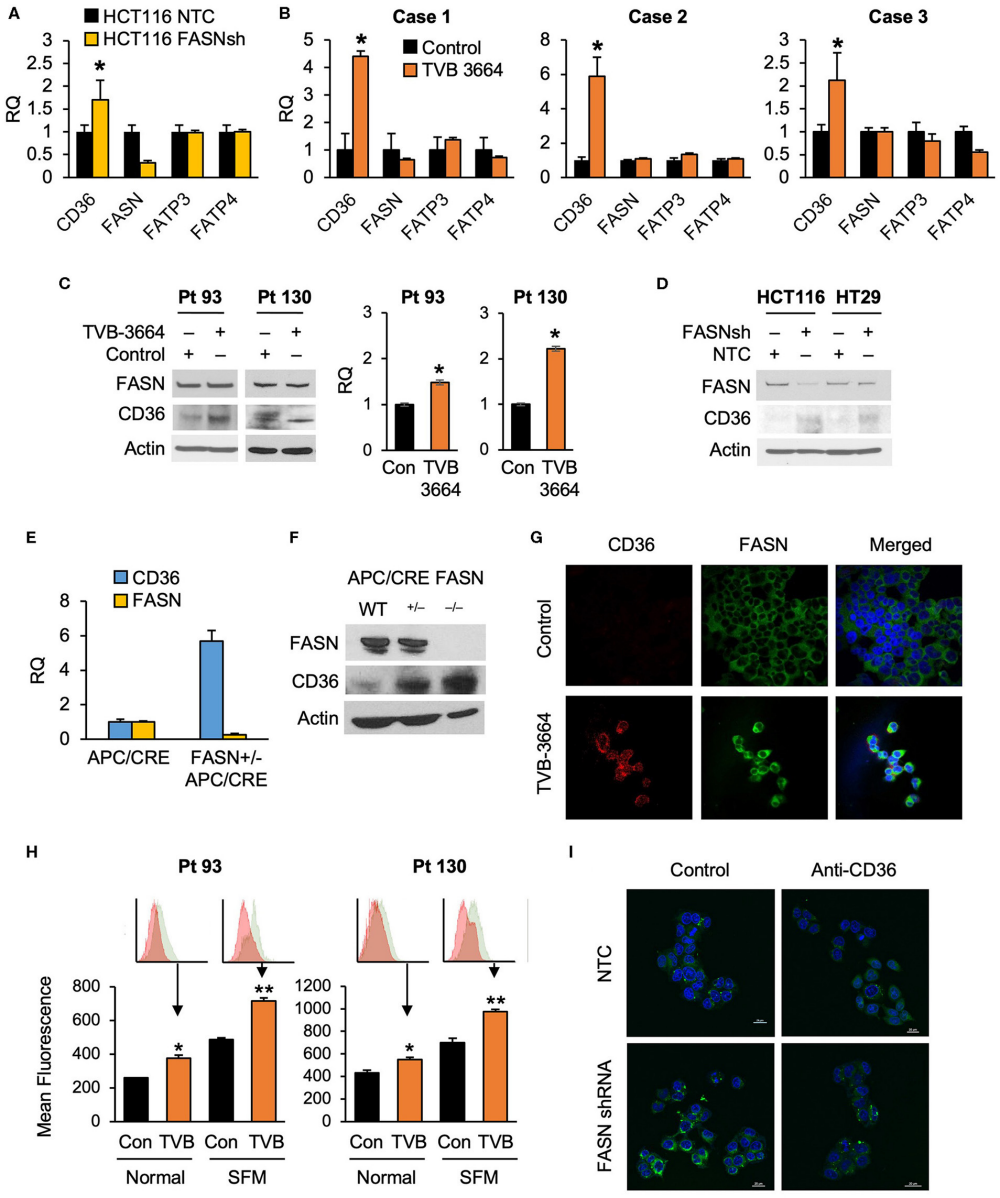
Expression of CD36 is selectively regulated by the level of *de novo* fatty acid synthesis in CRC. **(A)** shRNA-mediated knockdown of FASN leads to upregulation of CD36 mRNA expression in HCT116 cells (*p < 0.05). **(B)** TVB-3664 treatment of CRC tissue slices (18 h) selectively upregulates CD36 mRNA expression (*p < 0.05). **(C)** TVB-3664 treatment of Pt 93 and Pt 130 primary CRC cells increases CD36 mRNA and protein expression. **(D)** shRNA mediated knockdown of FASN increases CD36 protein expression in HCT116 and HT29 cells. **(E)** Relative mRNA expression of FASN and CD36 in intestinal tumors collected from APC/Cre and FASN^+/−^/APC/Cre mice. **(F)** FASN and CD36 protein expression in intestinal mucosa collected from Apc/Cre and Apc/Cre mice with hetero- and homo-zygous deletion of FASN. **(G,H)** Inhibition of FASN increases membrane-associated expression of CD36. **(G)** Confocal images of FASN and CD36 in control and 0.2 μM TVB-3664 treated (6 days) Pt 93 primary CRC cells. **(H)** Flow cytometry analysis of Pt 93 and Pt 130 primary CRC cells treated with 0.2 μM TVB-3664 (6 days) in normal and serum free media conditions. Mean fluorescence for CD36 is shown for representative data from three different experiments (**p < 0.01, *p < 0.05). **(I)** FA uptake in HCT116, NTC, and FASN shRNA. Cells were pre-treated with anti-CD36 antibody or vehicle for 24 h and then treated with BODIPY FL for 10 min.

To test whether alterations in FASN expression affect FA uptake, we assessed the expression of major FA transporters (FATPs and CD36) in HCT116 NTC and FASN shRNA CRC cells and found that FASN selectively upregulates mRNA expression of CD36, but not other FAs transporters (Figure 2A). To confirm that FASN selectively upregulates CD36, we treated fresh CRC human tissue slices with TVB-3664 and assessed the expression of FA transporters, including CD36. Consistent with our *in vitro* data, in all three CRC cases (Supplementary Table 1), we observed that CD36 mRNA expression increased at least two-fold and as much as four-fold when tissues were treated with TVB-3664. No changes were observed in expression of the other FA transporters tested (Figure 2B).

To further elucidate whether the level of endogenous fatty acid synthesis affects the expression of CD36, we next treated primary CRC cells from Pt 93 and Pt 130 with TVB-3664 for six days at a concentration of 0.2 μM as previously described (10). Inhibition of FASN led to an increase in CD36 mRNA and protein expression in both cell lines (Figure 2C). Consistently, shRNA-mediated knockdown of FASN in HCT116 and HT29 cell lines led to an increase in CD36 expression in normal and hypoxic conditions in both cell lines (Figure 2D, Supplementary Figure 2A). Interestingly, shRNA-mediated knockdown of CD36 does not affect FASN expression, suggesting a one-dimensional relationship between the two proteins (Supplementary Figure 2B).

The adenomatous polyposis coli (APC) gene product is mutated in the vast majority of human CRC and deletion of the APC gene leads to intestinal tumor formation in mice (33). In agreement with *in vitro* data, the analysis of intestinal tumors from mice with hetero- and homozygous deletions of FASN on C57BL/6-Apc/Cre background showed that deletion of FASN significantly upregulates CD36 expression (Figures 2E,F). Collectively, these data suggest that inhibition of FASN leads to selective upregulation of CD36 expression.

### Inhibition of FASN Leads to CD36 Translocation to Plasma Membrane

Confocal imaging of primary Pt 93 CRC cells, control, and treated with TVB-3664, shows that CD36 protein expression is upregulated and primarily localized to the plasma membrane when FASN is inhibited by TVB-3664 (Figure 2G). To confirm these data, primary CRC cells from Pt 93 and Pt 130 were treated with 0.2 μM TVB-3664 for 6 days in normal or serum-starved conditions and labeled with CD36-FITC antibody. Flow cytometry analysis was performed; the results confirmed that inhibition of FASN activity by TVB-3664 led to an increase in membrane-associated CD36 when compared to control cells in both cell lines in normal and serum-starved conditions (Figure 2H).

To confirm that this upregulation and translocation of CD36 to the plasma membrane was related to FA metabolism, a FA uptake assay was performed. HCT116, NTC, and FASN shRNA cells were plated and treated with BODIPY FL and imaged using confocal microscopy. We observed that FASN knockdown increases FA uptake as indicated by an increase in BODIPY FL staining (Figure 2I). Furthermore, to test that this increase in FAs within the cell was due to CD36 upregulation, we treated NTC and FASN shRNA cells with neutralizing antibody for CD36. As shown in Figure 2I, blocking CD36 has a minimum effect in NTC cells, but significantly decreases BODIPY FL uptake in FASN shRNA cells, further confirming that inhibition of FASN increases FA uptake via upregulation of CD36.

### Inhibition of CD36 Reduces CRC Cell Proliferation *in vitro*

**Figure 3:**
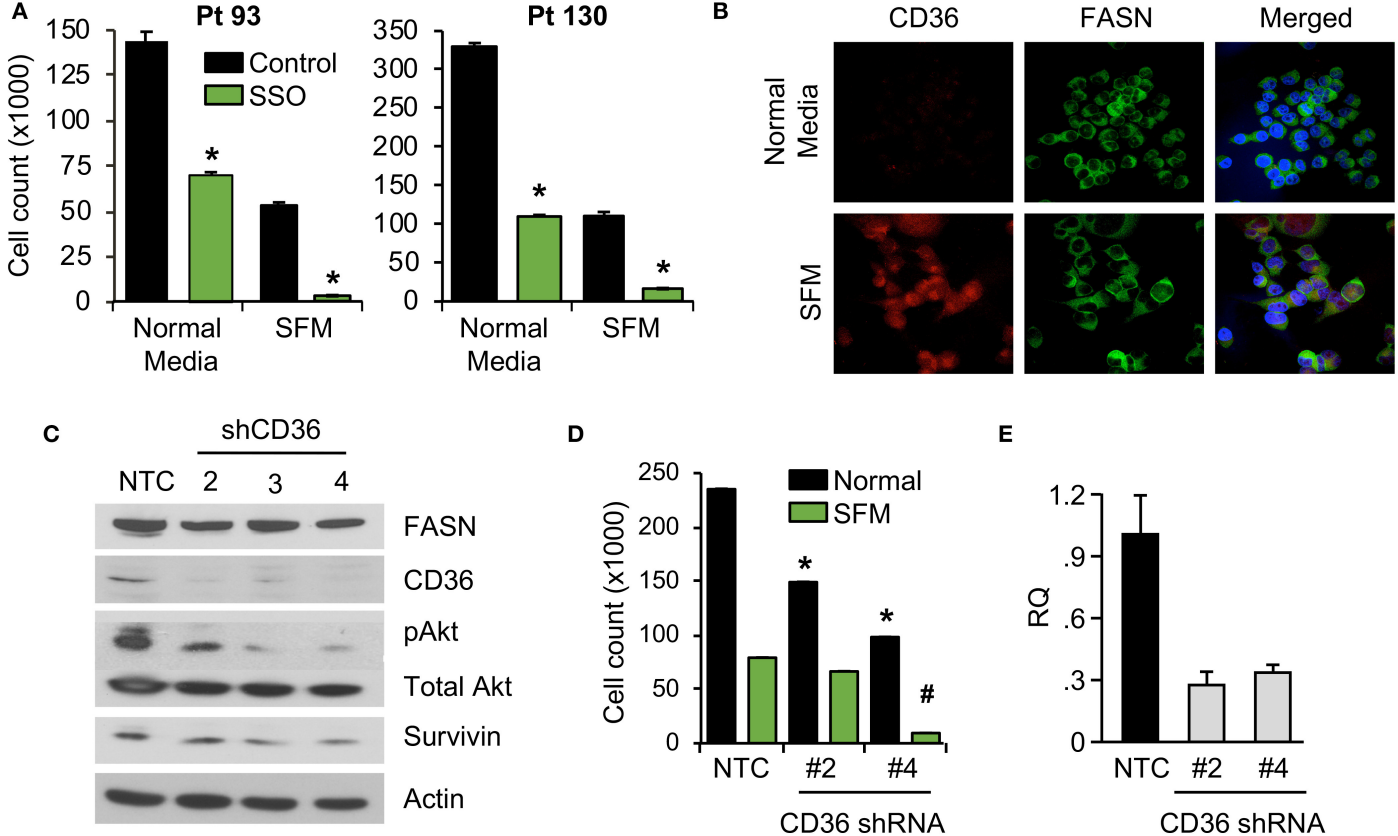
Inhibition of CD36 is associated with decreased cellular proliferation. **(A)** Primary Pt 93 and Pt 130 CRC cells treated with 100 μM SSO for 6 days. Cellular proliferation assays were performed via cell count. Representative data from three experiments is shown (*p < 0.05). **(B)** Confocal images of FASN and CD36 in Pt 93 cells in normal and serum free media (6 days). **(C)** Expression of proteins associated with apoptosis and survival in HCT116 transfected with CD36 shRNAs and analyzed via western blot. **(D)** Cellular proliferation assay with HCT116, NTC, and CD36 shRNA (*p < 0.05 for normal medium, ^#^*p* < for SFM). **(E)** qRT-PCR confirmation of CD36 knockdown using CD36 shRNA #2 (73%) and CD36 shRNA #4 (67%).

We have previously shown that stable knockdown and pharmacological inhibition of FASN are associated with a decrease in cellular proliferation and tumor growth (7, 10). However, the observed effects *in vivo* were not as prominent as the effects *in vitro*, suggesting the potential compensatory effects of diet and exogeneous FA uptake on tumor growth (7, 10). To test whether blocking fatty-acid uptake via CD36 has an effect on CRC cell proliferation, primary CRC cells, Pt 93 and Pt 130, were treated with the chemical CD36 inhibitor sulfosuccinimidyl oleate (SSO), which binds to CD36 via Lys164 in the hydrophobic cavity thereby impairing CD36-mediated fatty acid uptake (18, 34), at 100 μM in both normal and serum free medium (SFM) conditions. Under both conditions, primary CRC cells treated with SSO exhibited decreased cellular proliferation. Interestingly, sensitivity of both cell lines to SSO increased in SFM (Figure 3A). To evaluate differences in CD36 expression in normal and SFM, we performed confocal microscopy on Pt 93 cells cultured in normal and SFM conditions. We found that starvation of CRC cells leads to upregulation of CD36, which could explain an increase in sensitivity to SSO treatment (Figure 3B).

To assess the effect of CD36 overexpression on apoptotic markers we performed an Apoptosis Antibody Array. Data showed that overexpression of CD36 decreased caspase-3 cleavage and increased expression of survivin, a protein overexpressed in most transformed cell lines and malignancies and associated with poor clinical outcome (Supplementary Figure 3A) (27, 35). Consistently, western blot analysis of control and SSO-treated Pt 130 and Pt 93 primary CRC cells showed an increase in cleaved caspase-3 in both cell lines. A decrease in expression of survivin was observed in Pt 130 cells only (Supplementary Figure 3B). Consistent with pharmacological inhibition of CD36, shRNA-mediated knockdown of CD36 lead to a significant decrease in cellular proliferation and expression of survivin and pAkt in HCT116 cells (Figures 3C–E). Furthermore, shRNA-mediated knockdown of CD36 inhibits colony formation in the HT29 cell line (Supplementary Figure 3C). Together, these data demonstrate that CD36 promotes cellular proliferation in CRC.

### Inhibition and Knockdown of CD36 Reduces Xenograft Tumor Growth *in vivo*

**Figure 4:**
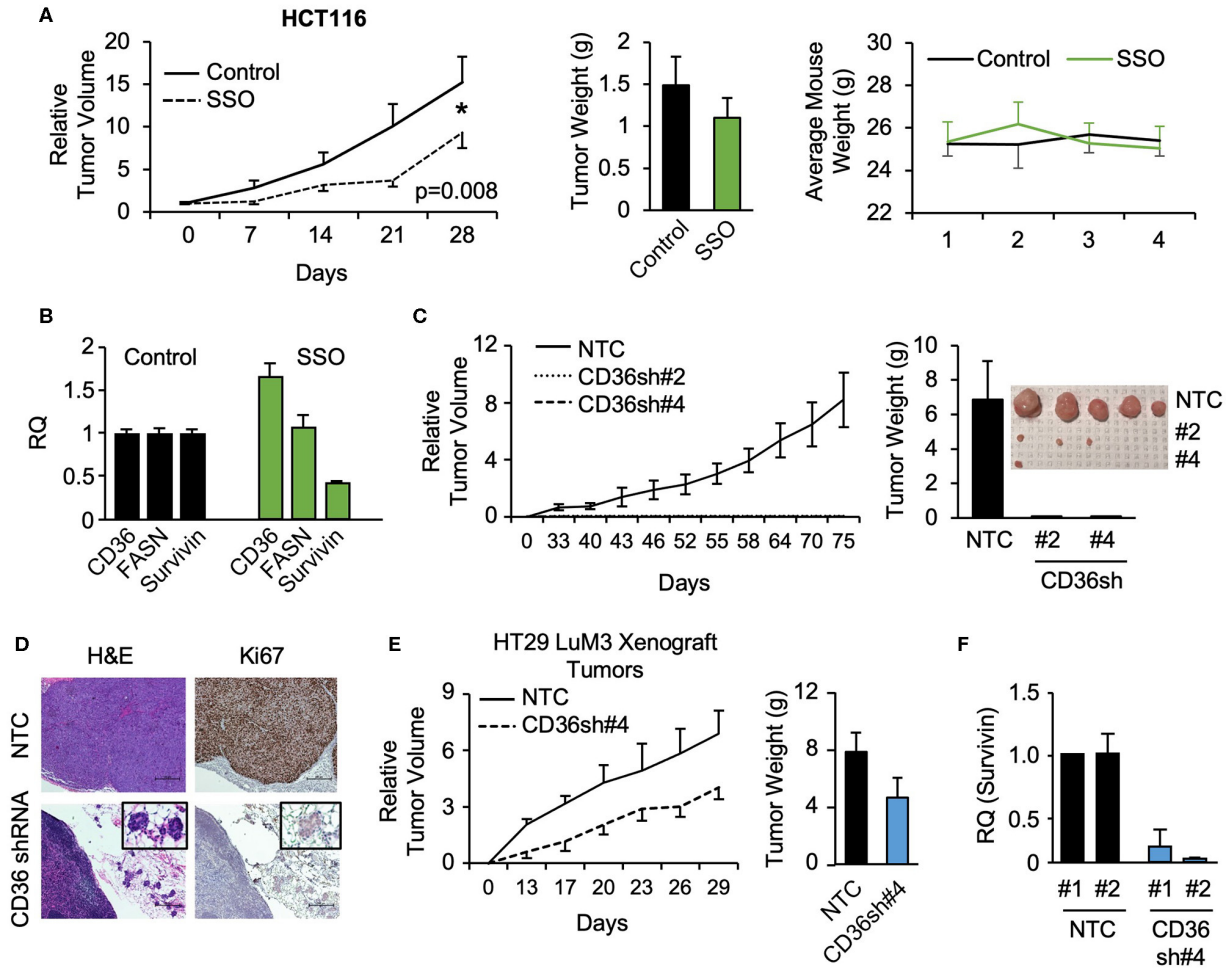
SSO treatment and CD36 knockdown inhibit tumor growth *in vivo*. **(A)** Tumor volume, tumor weight, and mouse weight of control and SSO treated (20 mg/kg) mice are shown. SSO was dissolved in 10% PEG and administered in 200 μl dosages via oral gavage daily. 1.0 × 10^6^ cells were injected into NU/NU mice. Treatment was initiated when tumors reached ~100 mm^3^ (day 0). **(B)** RT-PCR analysis of HCT116 tumors showing the effect of SSO treatment on CD36, FASN, and survivin mRNA expression. **(C)** Tumor volume of HCT116 NTC and CD36 shRNA #2 and #4 xenografts is shown. 1.0 × 10^6^ cells were injected into NU/NU mice and tumor growth was measured every 3 days. **(D)** H&E and Ki67 staining of HCT116 NTC and CD36 shRNA tumors. **(E)** Tumor volume and tumor weight of HT29 LuM3 NTC and CD36 shRNA #4 xenografts are shown. **(F)** mRNA expression of survivin in HT29 LuM3 xenografts (analysis of tumors from 2 mice per group is show).

To further investigate the role of CD36 in CRC tumor growth, HCT116 subcutaneous xenografts were treated with vehicle or SSO daily for 5 weeks. SSO treatment lead to significant decreases in tumor volume compared to vehicle control with no observable SSO toxicity as indicated by unchanged animal weight (Figure 4A). Furthermore, consistent with *in vitro* data, analysis of tumor tissues treated with SSO show a decrease in survivin mRNA (Figure 4B). FASN mRNA expression does not change with inhibition of CD36, further supporting the notion of a one directional relationship between the two proteins. Interestingly, SSO treatment led to an increase in CD36 mRNA suggesting that the potential compensation for the lack of functional CD36 was due to antagonistic action of SSO (Figure 4B).

To further investigate the role of CD36 in CRC tumor growth, the CRC cell lines, HCT116, HT29, and HT29 LuM3 [an HT29 cell line that was trained to efficiently metastasize to lung via *in vivo* selection process (36)], were established as subcutaneous xenografts. Interestingly, *in vivo* selection led to an increase in CD36 expression in HT29 LuM3 as compared to parental HT29 cells (Supplementary Figure 4A). HCT116, HT29, and HT29 LuM3 cells (NTC and shRNA-mediated CD36 knockdown cell lines) were injected subcutaneously into Nu/Nu mice and tumor growth was measured. Knockdown of CD36 in HCT116 cells markedly attenuated the growth of xenograft tumors compared to NTC (Figure 4C, Supplementary Figure 4B). In the case of CD36 shRNA #2 and CD36 shRNA #4 cells, we were able to identify microtumors at the site of injections. Tumor tissues were stained for Ki67, a known marker for tumor cell proliferation and growth (37). Ki67 expression was greatly reduced in CD36 knockdown tumors as compared to control (Figure 4D). In contrast to HCT116 cells, CD36 knockdown in HT29 did not significantly affect tumor growth, suggesting that this cell line may not be dependent on CD36 due to considerably lower CD36 expression as compared to HCT116 cells (Supplementary Figures 4A,C,D). However, CD36 knockdown using CD36 shRNA #4 in HT29 LuM3 cells, which have higher levels of CD36 expression as well as higher metastatic potential (36) (Supplementary Figure 4A), lead to a more prominent inhibition of tumor growth and a decrease in tumor weight (Figure 4E). Moreover, consistent with our *in vitro* data, qRT-PCR analysis of tumor tissues demonstrates a decrease in survivin expression when CD36 is knocked down in HT29 LuM3 tumors (Figure 4F). Thus, these data further support the role of CD36 in promoting CRC tumor growth.

### High Expression of CD36 Is Associated With an Increase in Survivin in CRC

**Figure 5:**
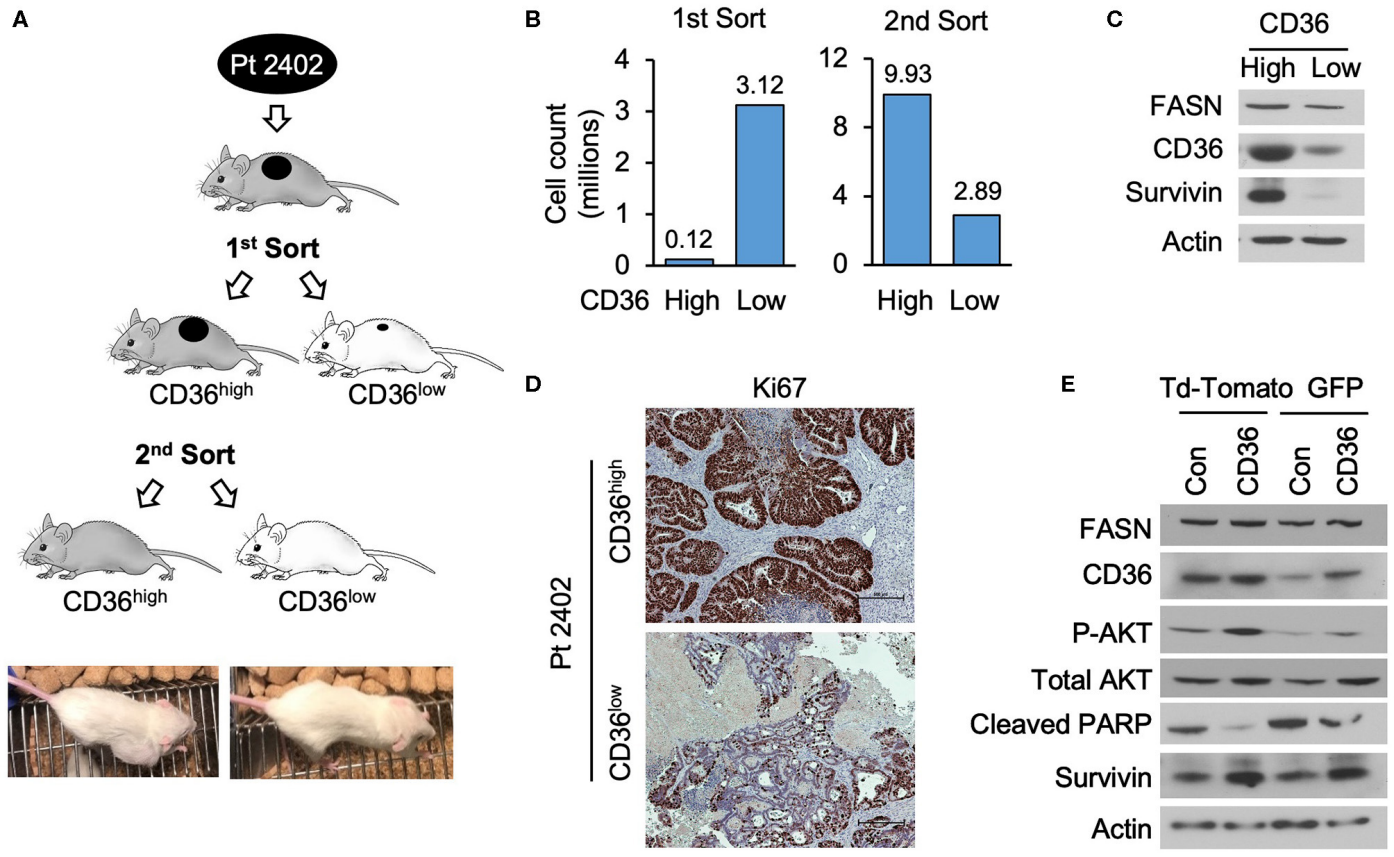
High expression of CD36 is associated with an increase in pAkt and survivin in CRC. **(A)** Diagram of Pt 2402 propagation after flow cytometry sorting for CD36^high^ and CD36^low^ cells. **(B)** Numbers of CD36^high^ and CD36^low^ Pt 2402 cells for first and second flow cytometry sorts. **(C)** Protein expression levels of FASN, CD36, and survivin in CD36^high^ and CD36^low^ Pt 2402 primary cells from first flow cytometry sort. **(D)** IHC staining for Ki67 in Pt 2402 CD36^high^ and CD36^low^ tumors. **(E)** Protein expression levels of FASN, CD36, pAkt, cleaved PARP, and survivin in HCT116 CRC cells, control, and CD36 overexpression.

To further establish that CD36 promotes cellular proliferation via upregulation of pro-survival pathways, we utilized a PDX tumor model, Pt 2402, which was established from a CRC metastasis to the lung (10) and is positive for CD36 expression (see Supplementary Figure 1C). Tumor tissue from first generation Pt 2402 PDX was inoculated into NOD/SCID mice and grown to ~1 cm^3^ volume. The tumor was excised, digested as previously described to a single cell suspension (29), stained with CD36-FITC and sorted via flow cytometry. The top 10% of the brightest green fluorescent protein (GFP) positive cells (117,000 cells), designated CD36^high^, and the bottom 10% of GFP negative cells (3,120,000 cells), designated CD36^low^, were sorted separately, placed in Matrigel, and sequentially implanted into NOD/SCID mice and allowed to grow. The tumor established from CD36^high^ cells grew much larger compared to the CD36^low^ tumor (tumor volume 2,419.64 vs. 53.57mm^3^, respectively; Figures 5A,B). Western blot analysis of tumor tissues from CD36^high^ and CD36^low^ cells showed an increase in survivin expression in the CD36^high^ tumors in comparison to the CD36^low^ tumors (Figure 5C). Interestingly, similar to our data obtained from TMA analysis, we observed that FASN was higher in CD36^high^ cells as compared to CD36^low^ cells, further supporting a potential interconnection between these two proteins (Figure 5C). Ki67 staining of Pt 2402 CD36^high^ and CD36^low^ tumors showed a significant reduction in Ki67 expression in the CD36^low^ tumors compared to CD36^high^ (Figure 5D).

To confirm that an increase in CD36 expression is associated with an increase in survivin expression, we overexpressed CD36 in the established HCT116 CRC cell line. Western blot analysis of HCT116 cells demonstrated CD36 overexpression leads to an increase in expression of survivin and activation of Akt, an upstream translational regulator of survivin in CRC (38), as well as a decrease in cleaved-PARP (Figure 5E). Therefore, taken together, our data suggest that upregulation of pAkt and survivin are potential mechanisms by which CD36 promotes CRC cell proliferation and tumor growth.

### Inhibition of FASN and CD36 in Combination Reduce Primary CRC Cell Proliferation *in vitro*

**Figure 6:**
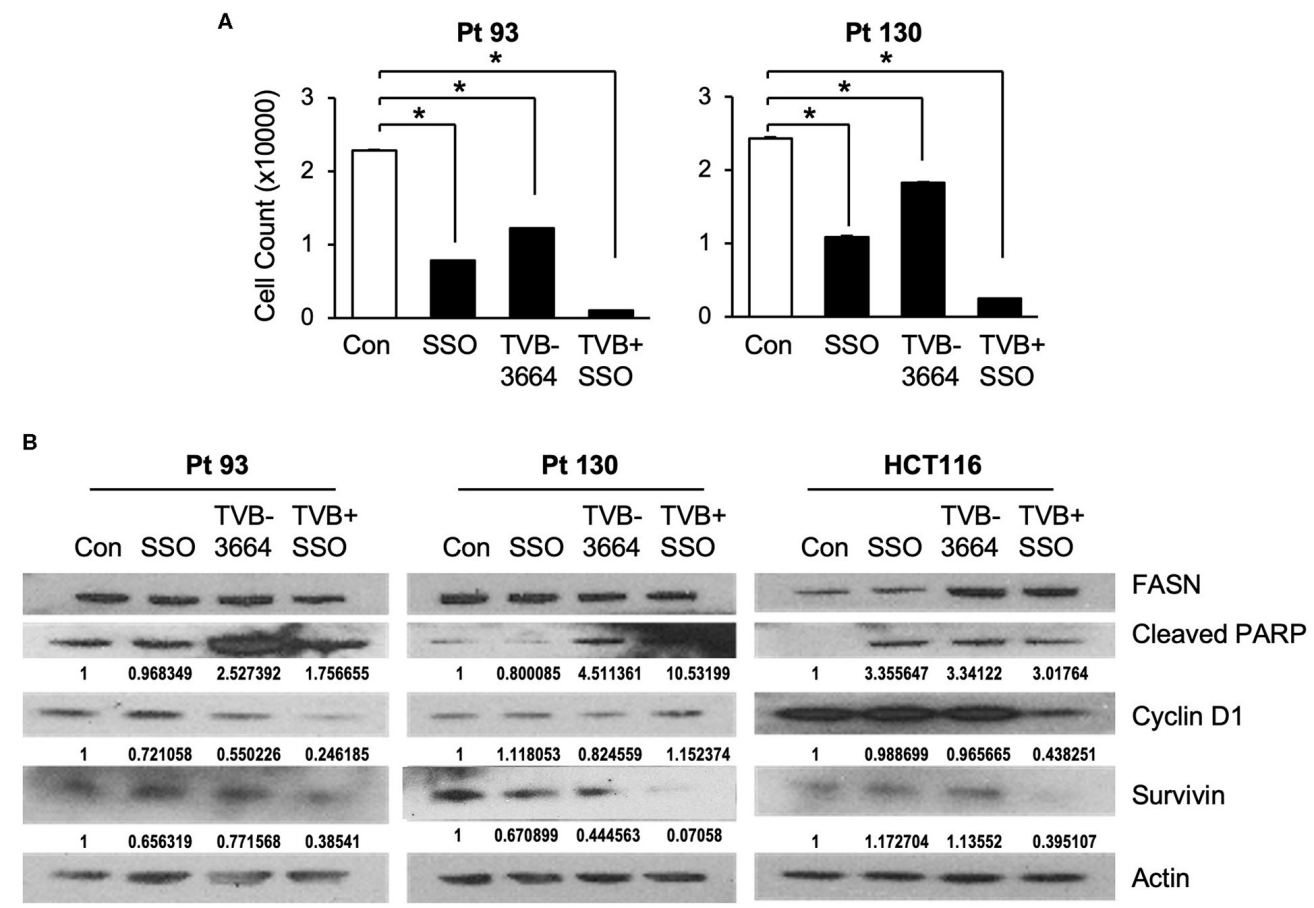
Inhibition of CD36 and FASN have a synergetic effect in reducing cell proliferation. **(A)** Pt 93, Pt 130, and HCT116 cells were treated with SSO and TVB-3664 alone or in combination for 6 days and cell number was counted. Representative data from three experiments is shown (*p < 0.05). **(B)** Western blot analysis of cells treated with TVB-3664, SSO, or TVB-3664 and SSO in combination.

Both *de novo* synthesized and exogenous FA play important roles in carcinogenesis (28, 39), To extend our findings that FASN inhibition upregulates the expression of CD36 and to further test whether inhibition of CD36 can improve the efficacy of TVB-3664, primary CRC cells from Pt 93 and Pt 130 were treated with CD36 inhibitor SSO and FASN inhibitor TVB-3664, alone or in combination, in both normal and serum-starved media. Cellular proliferation was significantly reduced in both SSO- and TVB-3664-treated cells and was further significantly reduced in cells that received combination treatment (Figure 6A). Western blot analysis of CRC cells treated with a combination of TVB-3664 and SSO shows that combination treatment significantly reduces expression of survivin in Pt 130 and HCT116 cell lines but not in Pt 93 cell line as compared to control or single agent treatment alone (Figure 6B). Combination treatment was also associated with reduced expression of cyclin D1 in Pt 93 and HCT116 cell lines. Interestingly, cyclin D1 in Pt 130 cells increased expression in combination treatments. This suggests a different mode of action and sensitivity to SSO and TVB-3664 in Pt 130 when compared to other CRC cell lines. Collectively, these data suggest that inhibition of both FA synthesis and FA uptake may be a potential therapeutic strategy for CRC. However, further studies are necessary to evaluate the effect of combinational treatment *in vivo*.

## Discussion

Our previous studies demonstrate that the effect of FASN inhibition on cellular proliferation *in vitro* does not always translate to the same effect on tumor growth *in vivo* (7, 10). Despite a significant decrease in cellular proliferation in primary CRC cell lines treated with TVB-3664, the efficacy of TVB-3664 in PDX models was much lower, suggesting a potential compensatory impact of diet on the effect of FASN inhibitors (10, 14). Therefore, the goal of this study was to delineate the effect of FASN inhibition on exogeneous FA uptake and elucidate the effect of FA uptake on sustaining cellular proliferation.

Here, for the first time, we report that inhibition of FASN leads to a selective upregulation of CD36 expression. CD36 enhances FA uptake and FA oxidation, and plays a critical role in cancer cell growth and metastasis (20, 21, 24, 40, 41). Consistent with reports that CD36 is upregulated in breast cancer and glioblastoma (42), we found that CD36 is highly expressed in CRC as compared to normal mucosa. Tumor stroma is deficient in CD36 expression (43). High stromal content in fresh primary CRC tumors can potentially explain why fresh CRC tissue analysis shows inconsistent results for CD36 expression in primary CRC as compare to normal colon. Based on tissue analysis, we found that high expression of CD36 is primarily associated with CRC metastasis, suggesting that metastatic tumors are more dependent on FA uptake as compared to primary CRC. Our findings are supported by multiple studies showing the involvement of CD36 in metastatic disease (24, 44, 45). Interestingly, even though we have identified a positive correlation between the protein expression of FASN and CD36 using TMA analysis, based on TCGA data, there is a significant inverse correlation between these two proteins at the mRNA levels. Indeed, we found that inhibition of FASN selectively upregulates CD36 mRNA and protein expression, but not expression of other FA transporters in multiple models including CRC cells, tumor xenografts, genetically modified mice and in human tissues. Interestingly, we did not note any significant changes in FASN expression when the expression of CD36 was altered, suggesting that the level of *de novo* lipid synthesis is not regulated by FA uptake via CD36 in our modes. Current understanding of the regulation of CD36 expression is rather limited (46), and how CD36 expression is regulated in cancer, and in particular in CRC, is not known. Several transcriptional activators have been implicated in regulation of CD36 expression including peroxisome proliferator-activated receptors (PPARs), CCAAT/enhancer-binding protein, and HIF-1 (16). Ongoing studies in our laboratory are investigating CD36 expression in different cell types in primary and metastatic CRC and potential mechanisms of CD36 regulation by FASN.

It has been reported that siRNA-mediated inhibition of CD36 decreases cellular proliferation in MCF-7 breast cancer cells (19). Additionally, CD36 has pro-tumorigenic and progression properties in glioblastoma stem cells (21). In agreement with these data, our study shows that chemical inhibition and stable knockdown of CD36 via shRNA in established and primary CRC cells decrease cellular proliferation. Consistent with data using a specific small molecule CD36 inhibitor, 2-methylthio-1,4-napthoquinone (MTN), in glioblastoma stem cells (21), inhibition of CD36 with SSO is associated with a decrease in activation of pAkt. We have also showed that CD36 regulates survivin, a member of the inhibitor of apoptosis (IAP) family that is highly expressed in most cancer and associated with a poor prognosis (47). The pro-survival role of CD36 in CRC is further supported by data showing that Pt 2402 CD36^high^ cells have a much higher propensity to establish xenograft tumors, which grow significantly faster and express higher levels of survivin, in comparison to CD36^low^ cells.

Interestingly, in a previously published study in oral carcinoma, the effect of CD36 inhibition was associated with inhibition of metastasis, but not with growth of primary oral cancers (24). In contrast to these findings, our study suggests a critical role of CD36 in CRC proliferation and tumor growth *in vivo* with both chemical inhibition via SSO as well as shRNA-mediated knockdown of CD36 in xenografts using multiple established cell lines.

Novel FASN inhibitors, TVBs, have demonstrated anticancer activity in multiple preclinical models (3), and TVB-2640 is currently in a number of clinical trials, including one at the University of Kentucky's Markey Cancer Center (https://www.cancer.gov/about-cancer/treatment/clinical-trials/search/v?id=NCI-2016-01710&r=1). Thus, it is crucial to identify and understand potential resistance mechanisms to FASN-targeted therapy. The current study demonstrates that inhibition of FASN leads to upregulation of CD36 expression and its translocation to the plasma membrane. One of the primary roles of CD36, when located within the cell membrane, is the transport of FAs (15, 16). Therefore, this upregulation of membrane bound CD36 and, consequently, an increase in FA uptake, could be a potential mechanism of resistance to FASN inhibition. Importantly, our data demonstrate that the combined inhibition of CD36 and FASN has a synergistic effect on inhibition of cellular proliferation as well as survivin and cyclin D1, further suggests that targeting FA uptake may be a potential therapeutic approach to increase the efficacy of FASN inhibitors.

We previously reported that the level of FASN expression determines the sensitivity of tumors to TVBs compounds (10). Consistently throughout this study, we observed that the higher expression of CD36 in HT29 LuM3 cells (36) as compared to parental HT29 cells, makes these cells more sensitive to CD36 inhibition via CD36 shRNA and inhibits xenograft tumor growth to a higher extent as compared to HT29 xenografts. Furthermore, the mutational and metabolic profiles of tumors determine tumor cell response to multiple therapies including metabolic inhibitors (48, 49). Different genetic profiles and metabolic features can explain the varying levels of response of cell lines to FASN and CD36 inhibition. Pt 93 and Pt 130 cells have KRAS and V600E BRAF mutations. The Pt 130 cell line also carries an FGFR mutation (10). The HCT116 cell line is a KRAS mutant, but BRAF wild type as compared to HT29 which has a V600E BRAF mutation but KRAS wild type (50). In addition, TVB-3664 seems to have more efficacy in activating PARP cleavage as compared to SSO, suggesting that inhibition of lipid synthesis leads to activation of apoptosis through distinct pathways other than those related to the inhibition of FA uptake. Our ongoing studies in the laboratory are focused on identifying the mutational and metabolic features of tumors that would determine their sensitivity to lipid metabolism targeted therapies.

Multiple studies suggest that fatty acid metabolism in adipose tissue is a major contributor to the etiology of obesity and diabetes (51). Obesity is associated with chronic elevation of free fatty acids, which promote insulin resistance and contribute to the development of systemic hyperglycemia (52). Interestingly, FASN expression is directly linked to obesity and type 2 diabetes (53) and CD36 protein expression is upregulated in both obese patients and type 2 diabetics (54). Therefore, the findings from this study support the idea that targeting both FASN and CD36 in combination may have therapeutic potential not only in cancer but also in metabolic disorders such as obesity and diabetes.

This report is the first to describe the functional importance of CD36 and its indolent role in fatty acid metabolism in the setting of CRC. It is also the first to describe the interconnection between FASN and CD36 and provides a strong rationale for further investigation into the interconnection of *de novo* lipogenesis and FA uptake that could potentially lead to the development of new therapeutic strategies for CRC and other solid malignancies, and potentially some metabolic disorders as well.

## Data Availability Statement

The raw data supporting the conclusions of this article will be made available by the authors, without undue reservation.

## Ethics Statement

The animal study was reviewed and approved by UK Animal Care and Use Committee, University of Kentucky. The studies involving human participants were reviewed and approved by UK Medical Center (IRB #16-0439-P2H), University of Kentucky. The patients/participants provided their written informed consent to participate in this study.

## Author Contributions

JD designed and performed experiments, wrote the methods, and drafted the manuscript. PR contributed to experimental design, data acquisition and interpretation, and reviewed the manuscript. NJ contributed to data acquisition. EL performed TMA scoring and tissue evaluation. HW performed statistical analysis. DH and CW performed analysis of TCGA data. BE contributed to experimental design and reviewed the manuscript. YZ contributed to experimental design, data acquisition and interpretation, and co-wrote the manuscript. All authors contributed to the article and approved the submitted version.

### Conflict of Interest

The authors declare that the research was conducted in the absence of any commercial or financial relationships that could be construed as a potential conflict of interest.

## References

[1] Ferlay J Shin HR Bray F Forman D Mathers C Parkin DM. Estimates of worldwide burden of cancer in 2008: GLOBOCAN (2008). Int J Cancer. (2010) 127:2893–917. 10.1002/ijc.25516 21351269

[2] Pavlova NN Thompson CB. The emerging hallmarks of cancer metabolism. Cell Metab. (2016) 23:27–47. 10.1016/j.cmet.2015.12.006 26771115 PMC4715268

[3] Buckley D Duke G Heuer TS O'Farrell M Wagman AS McCulloch W. Fatty acid synthase - modern tumor cell biology insights into a classical oncology target. Pharmacol Ther. (2017) 177:23–31. 10.1016/j.pharmthera.2017.02.021 28202364

[4] Currie E Schulze A Zechner R Walther TC Farese RV Jr. Cellular fatty acid metabolism and cancer. Cell Metab. (2013) 18:153–61. 10.1016/j.cmet.2013.05.017 23791484 PMC3742569

[5] Kim JT Liu C Zaytseva YY Weiss HL Townsend CM Jr Evers BM. Neurotensin, a novel target of Wnt/beta-catenin pathway, promotes growth of neuroendocrine tumor cells. Int J Cancer. (2015) 136:1475–81. 10.1002/ijc.29123 25098665 PMC4289421

[6] Zaytseva YY Elliott VA Rychahou P Mustain WC Kim JT Valentino J. Cancer cell-associated fatty acid synthase activates endothelial cells and promotes angiogenesis in colorectal cancer. Carcinogenesis. (2014) 35:1341–51. 10.1093/carcin/bgu042 24510238 PMC4043242

[7] Zaytseva YY Rychahou PG Gulhati P Elliott VA Mustain WC O'Connor K. Inhibition of fatty acid synthase attenuates CD44-associated signaling and reduces metastasis in colorectal cancer. Cancer Res. (2012) 72:1504–17. 10.1158/0008-5472.CAN-11-4057 22266115 PMC3596828

[8] Heuer TS Ventura R Mordec K Lai J Fridlib M Buckley D. FASN inhibition and taxane treatment combine to enhance anti-tumor efficacy in diverse xenograft tumor models through disruption of tubulin palmitoylation and microtubule organization and FASN inhibition-mediated effects on oncogenic signaling and gene expression. EBioMedicine. (2017) 16:51–62. 10.1016/j.ebiom.2016.12.012 28159572 PMC5474427

[9] Ventura R Mordec K Waszczuk J Wang Z Lai J Fridlib M. Inhibition of *de novo* palmitate synthesis by fatty acid synthase induces apoptosis in tumor cells by remodeling cell membranes, inhibiting signaling pathways, and reprogramming gene expression. EBioMedicine. (2015) 2:806–22. 10.1016/j.ebiom.2015.06.020 26425687 PMC4563160

[10] Zaytseva YY Rychahou PG Le AT Scott TL Flight RM Kim JT. Preclinical evaluation of novel fatty acid synthase inhibitors in primary colorectal cancer cells and a patient-derived xenograft model of colorectal cancer. Oncotarget. (2018) 9:24787–800. 10.18632/oncotarget.25361 29872506 PMC5973868

[11] O'Farrell M Crowley R Heuer TS Buckley D Rubino CM McCulloch W Abstract 2675: biomarker and PK/PD analyses of first in class FASN inhibitor TVB-2640 in a first-in-human phase 1 study in solid tumor patients. AACR. (2015) 75(Suppl):2675 10.1158/1538-7445.AM2015-2675

[12] Dean EJ Falchook GS Patel MR Brenner AJ Infante JR Arkenau H-T Abstract 2512: Preliminary activity in the first in human study of the first-in-class fatty acid synthase (FASN) inhibitor, TVB-2640. J Clin Oncol. (2016) 34(15_suppl):2512 10.1200/JCO.2016.34.15_suppl.2512

[13] National Cancer Institute FASN Inhibitor TVB-2640 in Treating Patients with Colon or Other Cancers That Can Be Removed by Surgery. National Institutes of Health (2017). Available online at: https://www.cancer.gov/about-cancer/treatment/clinical-trials/search/v?id=NCI-2016-01710&r=1

[14] Jafari N Drury J Morris AJ Onono FO Stevens PD Gao T. *De novo* fatty acid synthesis-driven sphingolipid metabolism promotes metastatic potential of colorectal cancer. Mol Cancer Res. (2019) 17:140–52. 10.1158/1541-7786.MCR-18-0199 30154249 PMC6318071

[15] Pepino MY Kuda O Samovski D Abumrad NA. Structure-function of CD36 and importance of fatty acid signal transduction in fat metabolism. Annu Rev Nutr. (2014) 34:281–303. 10.1146/annurev-nutr-071812-161220 24850384 PMC4329921

[16] Glatz JFC Luiken J. Dynamic role of the transmembrane glycoprotein CD36 (SR-B2) in cellular fatty acid uptake and utilization. J Lipid Res. (2018) 59:1084–93. 10.1194/jlr.R082933 29627764 PMC6027920

[17] Glatz JF Luiken JJ Bonen A. Membrane fatty acid transporters as regulators of lipid metabolism: implications for metabolic disease. Physiol Rev. (2010) 90:367–417. 10.1152/physrev.00003.2009 20086080

[18] Luiken JJ Chanda D Nabben M Neumann D Glatz JF. Post-translational modifications of CD36 (SR-B2): implications for regulation of myocellular fatty acid uptake. Biochim Biophys Acta. (2016) 1862:2253–8. 10.1016/j.bbadis.2016.09.004 27615427

[19] Zhao J Zhi Z Wang C Xing H Song G Yu X. Exogenous lipids promote the growth of breast cancer cells via CD36. Oncol Rep. (2017) 38:2105–15. 10.3892/or.2017.5864 28765876 PMC5652970

[20] Ladanyi A Mukherjee A Kenny HA Johnson A Mitra AK Sundaresan S. Adipocyte-induced CD36 expression drives ovarian cancer progression and metastasis. Oncogene. (2018) 37:2285–301. 10.1038/s41388-017-0093-z 29398710 PMC5920730

[21] Hale JS Otvos B Sinyuk M Alvarado AG Hitomi M Stoltz K. Cancer stem cell-specific scavenger receptor CD36 drives glioblastoma progression. Stem Cells. (2014) 32:1746–58. 10.1002/stem.1716 24737733 PMC4063873

[22] Pan J Fan Z Wang Z Dai Q Xiang Z Yuan F. CD36 mediates palmitate acid-induced metastasis of gastric cancer via AKT/GSK-3β/β-catenin pathway. J Exp Clin Cancer Res. (2019) 38:52. 10.1186/s13046-019-1049-7 30717785 PMC6360779

[23] Watt MJ Clark AK Selth LA Haynes VR Lister N Rebello R. Suppressing fatty acid uptake has therapeutic effects in preclinical models of prostate cancer. Sci Transl Med. (2019) 11:eaau5758. 10.1126/scitranslmed.aau5758 30728288

[24] Pascual G Avgustinova A Mejetta S Martin M Castellanos A Attolini CS. Targeting metastasis-initiating cells through the fatty acid receptor CD36. Nature. (2017) 541:41–5. 10.1038/nature20791 27974793

[25] Menendez JA Lupu R. Fatty acid synthase and the lipogenic phenotype in cancer pathogenesis. Nat Rev Cancer. (2007) 7:763–77. 10.1038/nrc2222 17882277

[26] Altieri DC. Targeting survivin in cancer. Cancer Lett. (2013) 332:225–8. 10.1016/j.canlet.2012.03.005 22410464 PMC3695618

[27] Garg H Suri P Gupta JC Talwar GP Dubey S. Survivin: a unique target for tumor therapy. Cancer Cell Int. (2016) 16:49. 10.1186/s12935-016-0326-1 27340370 PMC4917988

[28] DeBerardinis RJ Chandel NS. Fundamentals of cancer metabolism. Sci Adv. (2016) 2:e1600200. 10.1126/sciadv.1600200 27386546 PMC4928883

[29] Jafari N Drury J Morris AJ Onono FO Stevens PD Gao T *De novo* fatty acid synthesis driven sphingolipid metabolism promotes metastatic potential of colorectal cancer. Mol Cancer Res. (2018) 78(Suppl):1437 10.1158/1538-7445.AM2018-1437 PMC6318071 30154249

[30] Uhlen M Fagerberg L Hallstrom BM Lindskog C Oksvold P Mardinoglu A. Proteomics. Tissue-based map of the human proteome. Science. (2015) 347:1260419. 10.1126/science.1260419 25613900

[31] Silverstein RL Febbraio M. CD36, a scavenger receptor involved in immunity, metabolism, angiogenesis, and behavior. Sci Signal. (2009) 2:re3. 10.1126/scisignal.272re3 19471024 PMC2811062

[32] Julien S Merino-Trigo A Lacroix L Pocard M Goere D Mariani P. Characterization of a large panel of patient-derived tumor xenografts representing the clinical heterogeneity of human colorectal cancer. Clin Cancer Res. (2012) 18:5314–28. 10.1158/1078-0432.CCR-12-0372 22825584

[33] Cheung AF Carter AM Kostova KK Woodruff JF Crowley D Bronson RT. Complete deletion of Apc results in severe polyposis in mice. Oncogene. (2010) 29:1857–64. 10.1038/onc.2009.457 20010873 PMC2990498

[34] Coort SL Willems J Coumans WA van der Vusse GJ Bonen A Glatz JF. Sulfo-N-succinimidyl esters of long chain fatty acids specifically inhibit fatty acid translocase (FAT/CD36)-mediated cellular fatty acid uptake. Mol Cell Biochem. (2002) 239:213–9. 10.1007/978-1-4419-9270-3_27 12479588

[35] Sommer KW Schamberger CJ Schmidt GE Sasgary S Cerni C. Inhibitor of apoptosis protein (IAP) survivin is upregulated by oncogenic c-H-Ras. Oncogene. (2003) 22:4266–80. 10.1038/sj.onc.1206509 12833149

[36] Rychahou P Bae Y Reichel D Zaytseva YY Lee EY Napier D. Colorectal cancer lung metastasis treatment with polymer-drug nanoparticles. J Control Release. (2018) 275:85–91. 10.1016/j.jconrel.2018.02.008 29421609 PMC5908241

[37] Sahin AA Ro JY Brown RW Ordonez NG Cleary KR el-Naggar AK. Assessment of Ki-67-derived tumor proliferative activity in colorectal adenocarcinomas. Mod Pathol. (1994) 7:17–22. 8159647

[38] Ye Q Cai W Zheng Y Evers BM She QB. ERK and AKT signaling cooperate to translationally regulate survivin expression for metastatic progression of colorectal cancer. Oncogene. (2014) 33:1828–39. 10.1038/onc.2013.122 23624914 PMC3966979

[39] Cao D Song X Che L Li X Pilo MG Vidili G. Both *de novo* synthetized and exogenous fatty acids support the growth of hepatocellular carcinoma cells. Liver Int. (2017) 37:80–9. 10.1111/liv.13183 27264722 PMC5140766

[40] Cheng C Geng F Cheng X Guo D. Lipid metabolism reprogramming and its potential targets in cancer. Cancer Commun (Lond). (2018) 38:27. 10.1186/s40880-018-0301-4 29784041 PMC5993136

[41] Clezardin P Frappart L Clerget M Pechoux C Delmas PD. Expression of thrombospondin (TSP1) and its receptors (CD36 and CD51) in normal, hyperplastic, and neoplastic human breast. Cancer Res. (1993) 53:1421–30. 7680285

[42] Liang Y Han H Liu L Duan Y Yang X Ma C. CD36 plays a critical role in proliferation, migration and tamoxifen-inhibited growth of ER-positive breast cancer cells. Oncogenesis. (2018) 7:98. 10.1038/s41389-018-0107-x 30573731 PMC6302092

[43] Wang J Li Y. CD36 tango in cancer: signaling pathways and functions. Theranostics. (2019) 9:4893–908. 10.7150/thno.36037 31410189 PMC6691380

[44] Enciu AM Radu E Popescu ID Hinescu ME Ceafalan LC. Targeting CD36 as biomarker for metastasis prognostic: how far from translation into clinical practice? Biomed Res Int. (2018) 2018:7801202. 10.1155/2018/7801202 30069479 PMC6057354

[45] Pascual G Dominguez D Benitah SA. The contributions of cancer cell metabolism to metastasis. Dis Model Mech. (2018) 11:dmm032920. 10.1242/dmm.032920 29739810 PMC6124557

[46] Showkat M Beigh MA Andrabi KI. mTOR signaling in protein translation regulation: implications in cancer genesis and therapeutic interventions. Mol Biol Int. (2014) 2014:686984. 10.1155/2014/686984 25505994 PMC4258317

[47] Altieri DC. Survivin, cancer networks and pathway-directed drug discovery. Nat Rev Cancer. (2008) 8:61–70. 10.1038/nrc2293 18075512

[48] Luengo A Gui DY Vander Heiden MG. Targeting metabolism for cancer therapy. Cell Chem Biol. (2017) 24:1161–80. 10.1016/j.chembiol.2017.08.028 28938091 PMC5744685

[49] Parca L Pepe G Pietrosanto M Galvan G Galli L Palmeri A. Modeling cancer drug response through drug-specific informative genes. Sci Rep. (2019) 9:15222. 10.1038/s41598-019-50720-0 31645597 PMC6811538

[50] Ahmed D Eide PW Eilertsen IA Danielsen SA Eknaes M Hektoen M. Epigenetic and genetic features of 24 colon cancer cell lines. Oncogenesis. (2013) 2:e71. 10.1038/oncsis.2013.35 24042735 PMC3816225

[51] Park J Morley TS Kim M Clegg DJ Scherer PE. Obesity and cancer–mechanisms underlying tumour progression and recurrence. Nat Rev Endocrinol. (2014) 10:455–65. 10.1038/nrendo.2014.94 24935119 PMC4374431

[52] Hellmann J Zhang MJ Tang Y Rane M Bhatnagar A Spite M. Increased saturated fatty acids in obesity alter resolution of inflammation in part by stimulating prostaglandin production. J Immunol. (2013) 191:1383–92. 10.4049/jimmunol.1203369 23785121 PMC3720716

[53] Berndt J Kovacs P Ruschke K Kloting N Fasshauer M Schon MR. Fatty acid synthase gene expression in human adipose tissue: association with obesity and type 2 diabetes. Diabetologia. (2007) 50:1472–80. 10.1007/s00125-007-0689-x 17492427

[54] Balaban S Lee LS Schreuder M Hoy AJ. Obesity and cancer progression: is there a role of fatty acid metabolism? Biomed Res Int. (2015) 2015:274585. 10.1155/2015/274585 25866768 PMC4383231

