# Supplementary material for: Inhibition of Fatty Acid Synthase Upregulates Expression of CD36 to Sustain Proliferation of Colorectal Cancer Cells
Source: Front Oncol. 2020 Jul 31;10:1185. doi: 10.3389/fonc.2020.01185 (PMC7411002; doi:10.3389/fonc.2020.01185)
Supplement: Supplementary file 1 [file Table_1.pdf]

Supplementary Table 1. Case Characteristics.

Case	Gender	Age	Pathology	Operation	Staging
Case 1	M	66	Invasive moderately differentiated adenocarcinoma	Low anterior colon resection	T3N1a
Case 2	F	90	Invasive moderately differentiated colorectal adenocarcinoma	Left hemicolectomy	T3N0
Case 3	F	68	Invasive adenocarcinoma involving muscularis propria	Right hemicolectomy	T2N0
