# Supplementary material for: Inhibition of Fatty Acid Synthase Upregulates Expression of CD36 to Sustain Proliferation of Colorectal Cancer Cells
Source: Front Oncol. 2020 Jul 31;10:1185. doi: 10.3389/fonc.2020.01185 (PMC7411002; doi:10.3389/fonc.2020.01185)
Supplement: Supplementary file 2 [file Data_Sheet_1.docx]

**
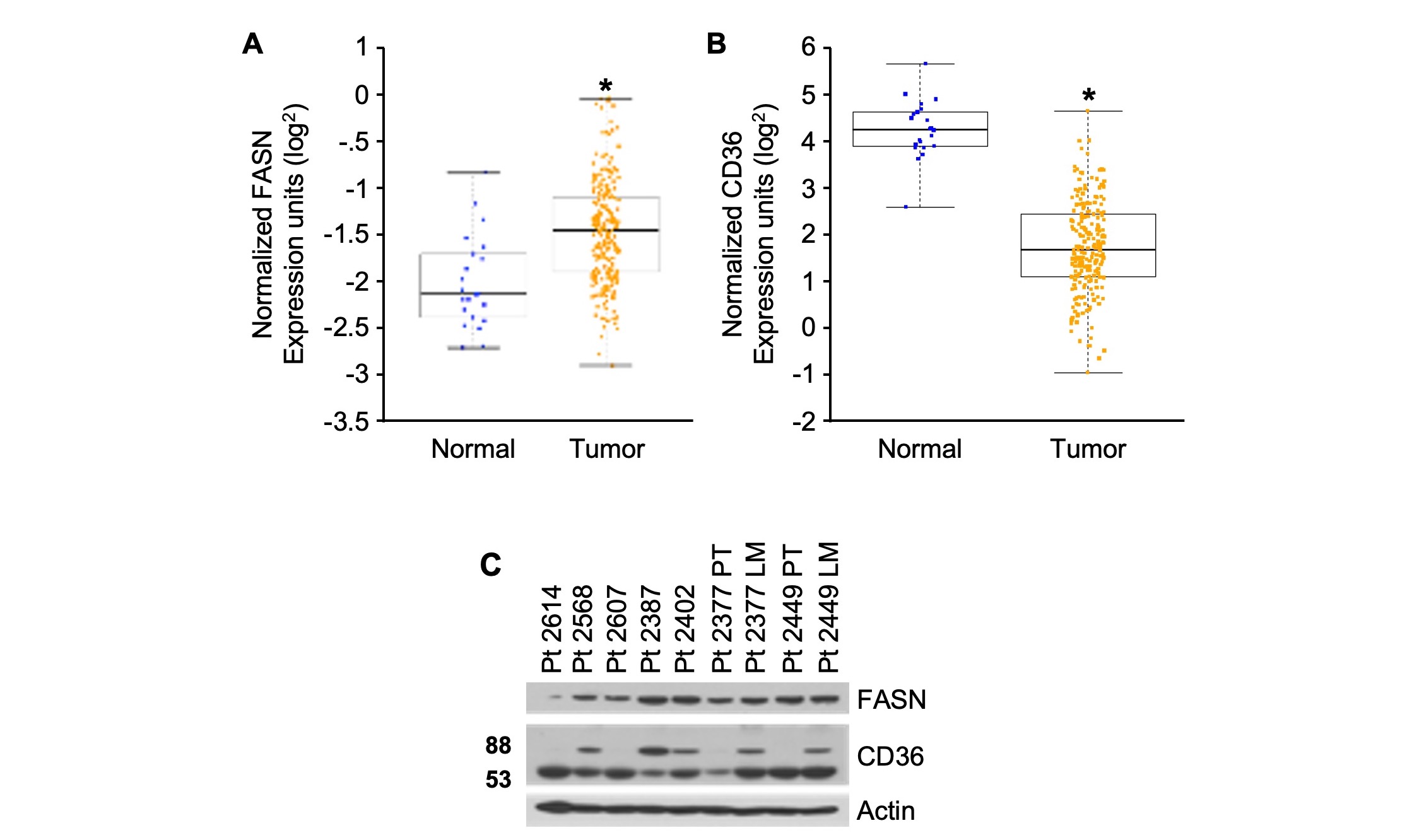
**

**Supplementary Figure 1. (A)** FASN mRNA expression is increased (**p* < 0.0001) and **(B)** CD36 mRNA expression is decreased (**p* < 0.0001) in CRC patient samples in the TCGA dataset (n=22 of normal tissues and n=215 of tumors). **(C)** Expression of FASN and CD36 in PDX tumor tissues.

**
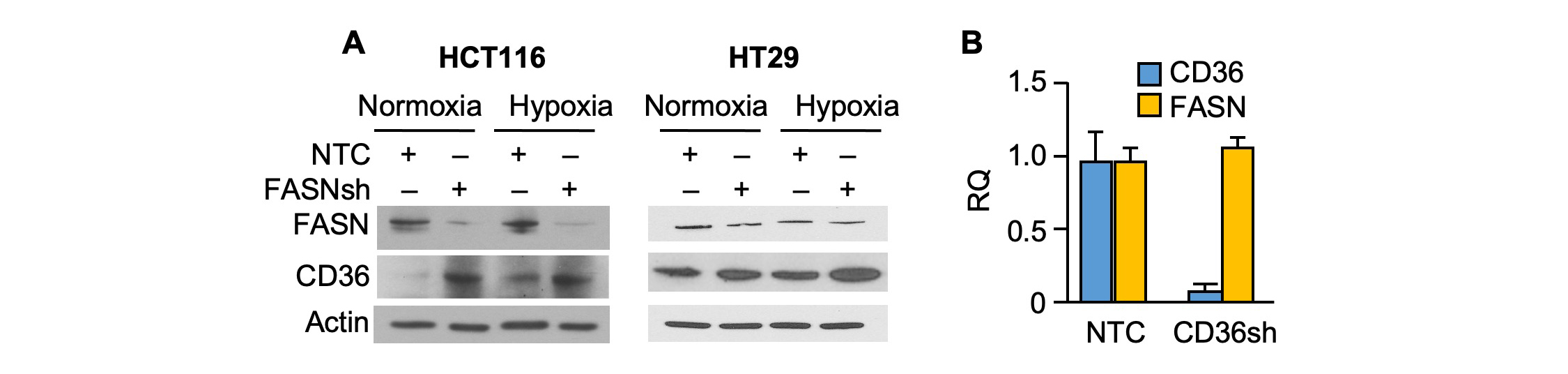
**

**Supplementary Figure 2.** Expression of CD36 is regulated by the level of *de novo* fatty acid synthesis in CRC. **(A)** Protein expression of CD36 and FASN in FASN shRNA HCT116 and HT29 cells in both normal and hypoxic conditions. **(B)** mRNA expression of CD36 and FASN in HT29LM3 NTC and CD36 shRNA.

**
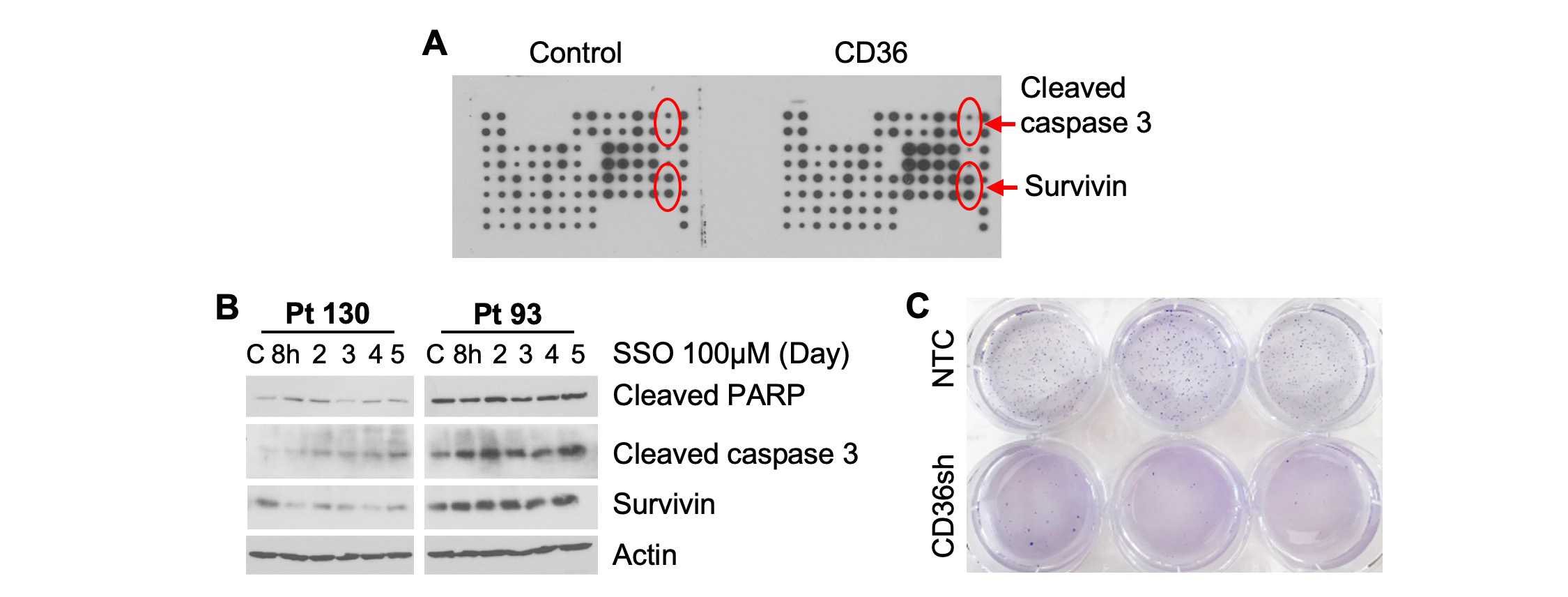
**

**Supplementary Figure 3.** CD36 upregulates survivin and inhibits apoptotic markers. **(A)** Effect of CD36 overexpression on apoptotic markers was assessed in HCT116 cells, control and tdTomato-CD36, using Apoptosis Antibody Array. **(B)** Primary Pt 130 and Pt 93 CRC cells were treated with 100μM SSO for 5 days in normal medium and expression of cleaved PARP, cleaved caspase-3, and survivin was analyzed. **(C)** 3 x 10^4^ HT29, NTC and CD36 shRNA #4, cells were plated in agarose and allowed to grow for 10 days.


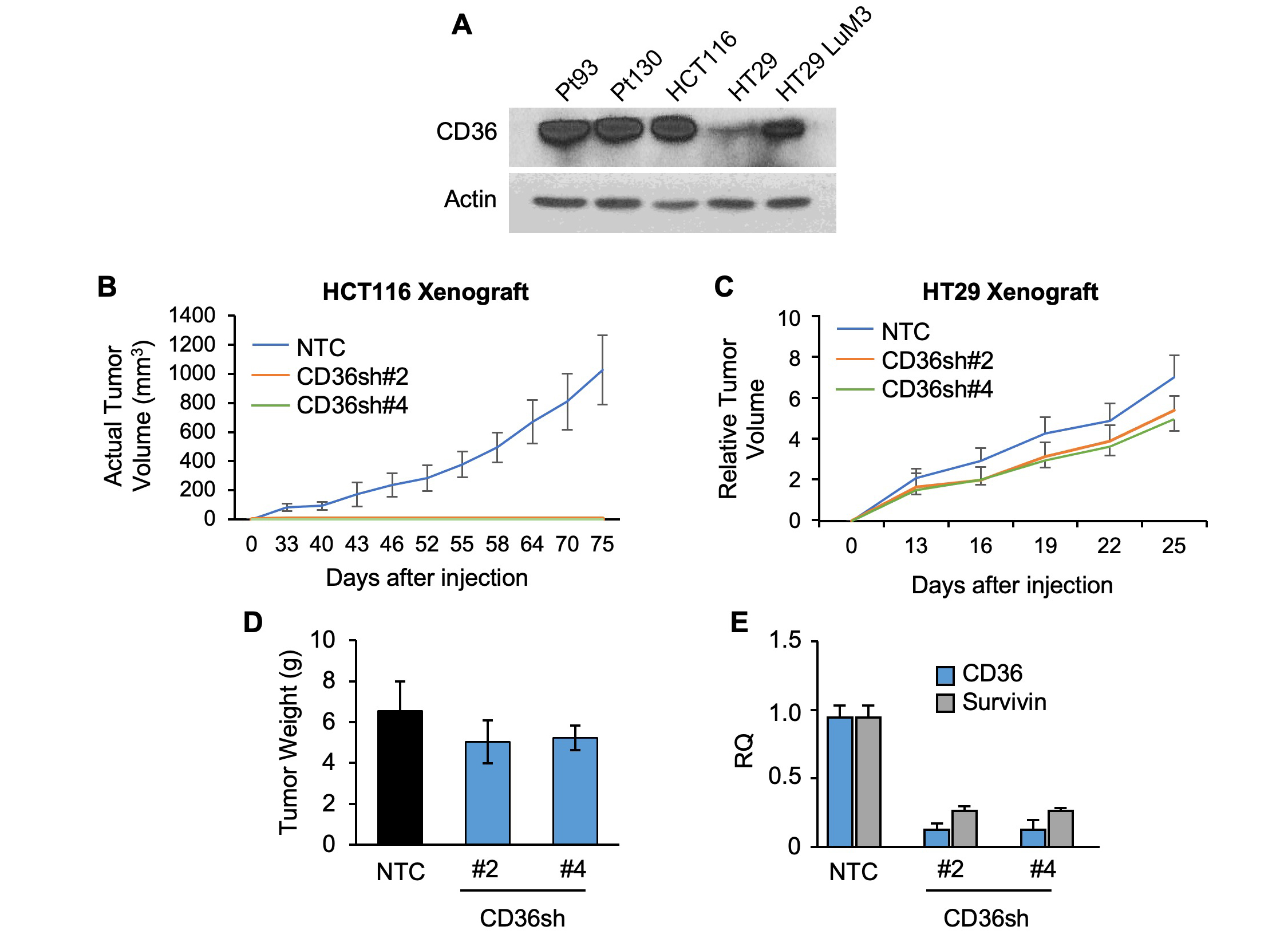


**Supplementary Fig. 4. (A)** Expression of CD36 in CRC cell lines. **(B)** Tumor volume (mm^3^) of HCT116, NTC and CD36 shRNA#2 and #4 xenografts shown in Figure 4C. **(C)** Tumor growth for HT29 NTC and CD36 shRNA #2 and #4 xenografts. 2.0 x 10^6^ cells were injected into NU/NU mice and tumor growth was measured every 3 days. **(D)** Tumor weight for HT29 NTC and CD36 shRNA #2 and #4 xenografts. **(E)** mRNA expression of CD36 and survivin in HT29 xenograft tumors.
